# On collective behavior in *C. elegans*

**DOI:** 10.3389/fnbot.2025.1689332

**Published:** 2025-11-17

**Authors:** Nemanja Antonic, Aymeric Vellinger, Elio Tuci

**Affiliations:** Faculty of Computer Science, University of Namur, Namur, Belgium

**Keywords:** *C. elegans*, aggregation, collective decision-making, swarming, collective behavior

## Abstract

*C. elegans* is a model organism in many biological domains, such as genetics, neurophysiology, and behavioral ecology. Despite our relatively deep knowledge of the neuronal, genetic and molecular mechanisms underlying *C. elegans* communication, we still lack a comprehensive understanding of emergent group-level dynamics. We review the literature on collective behavior of *C. elegans* by categorizing works in this relatively small research field along three main axes corresponding to primary collective responses: aggregation, swarming, and collective decision-making. Through an analysis of the methods and scientific contributions of these works, we develop a critical perspective that points to important gaps in our understanding of the mechanisms underlaying the emergence of collective responses. We discuss the consequences of the lack of evidence concerning the effect of population density on the emergence of specific group dynamics, and the relatively limited knowledge related to how self-generated pheromones regulate local interactions and contribute to the emergence of group responses. We elaborate on the methodological problems of developing experimental scenarios to disentangle causal relationships between population density, pheromone-based interactions and collective responses. We propose to overcome these limitations with an interdisciplinary approach based on the use of *in vivo* experiments, mathematical and computer-based models.

## 1 Introduction

In biology, many different species carry out activities engaging multiple individuals. These activities are generally referred to as collective behavior, such as flocking in birds, schooling in fishes, and herding in different mammals. Complex forms of collective behavior can also be observed in social insects, such as ants, bees, and termites, where important decisions about where to search for food or where to build the nest are made collectively (see e.g., Bonabeau et al., 1997; Seeley and Buhrman, 1999).

The combined results of studies based on the direct observation of collective behavior in both natural habitats and laboratory settings revealed that distributed individual mechanisms underpin coordinated and collaborative activity. In other words, group responses emerge as a spontaneous (self-organized) spatio-temporal order from repeated individual interactions generally governed by simple rules (Sumpter, 2006; Camazine et al., 2020).

In the last two decades, self-organized collective behaviors in natural organisms have inspired roboticists who have sought to engineer into robots local individual rules underpinning self-organized group responses (Schranz et al., 2021). The most emblematic example of this interdisciplinary approach to technology building is swarm robotics, a domain in which robots are: (i) designed by taking into account some of the most salient characteristics of social insects, such as distributed control, local perception, and simple stigmergic-like forms of communication (Holland and Melhuish, 1999) and (ii) programmed to react locally in order to trigger the virtuous self-organized processes leading to complex group responses (Dorigo and Şahin, 2004; Brambilla et al., 2013). Bioinspired principles provide swarm roboticists a promising approach for creating robots capable of displaying adaptivity, robustness to changing environmental conditions, resilience and fault-tolerance similar to that observed in social insects (Trianni and Campo, 2015). However, several issues are still severely limiting the deployment of swarms of robots outside laboratory settings in natural, unconstrained environments (Dorigo et al., 2021). Some of these issues are inherently linked to theoretical and methodological limitations in engineering self-organized collective behavior in robots. Other limiting issues are those common to all electro-mechanical and silicon-based artificial systems which lack the perceptual acuity, dexterity, adaptability and resilience that make biological systems particularly suited to operate in their respective ecological niches.

Constant scientific progress pushes further the boundaries of knowledge and consequently fuels innovation and technological advancement in robotics. Very recently a radically different approach to the design of robots has emerged, one which calls for biological animal robots; that is, biological organisms that are behaviorally reprogrammed through genetic modifications to behave not as they would according to their evolutionary and ontogenetic history, but as desired by humans (Rabinowitch, 2019a). The idea of biological animal robots is made possible by synthetic connectomics (Rabinowitch, 2019b) a discipline consisting on the modification of an organism neural layout as described in its connectome (i.e., a map of the neural connections). This can be done through the genetic engineering of new synaptic connections (Rabinowitch et al., 2024), together with the implementation of additional synthetic biological tools (Kukhtar and Fussenegger, 2023).

The first animal whose complete connectome had been mapped out is *C. elegans*, a 1 mm roundworm with 302 neurons (White et al., 1986) making it an ideal candidate for the application of synaptic and neuronal engineering. The methods of synthetic connectomics can be used to instill specific human-desired behaviors in *C. elegans* by equipping the worm with a connectome designed to induce a particular response in a completely autonomous fashion. Swarms of biological animal robots could potentially be built using populations of *C. elegans* with synthetically restructured connectomes, bringing forth specific sequences of emergent group responses (e.g., aggregation, swarming toward a target, collective decisions, coordinated, and cooperative actions, etc.) (Rabinowitch, 2019a).

The first step in applying synaptic and neuronal engineering to *C. elegans* is to understand the operational principles underlying behavioral responses, which helps to define the building blocks that will eventually be modified to create worms with a partially or completely artificial connectome. The majority of the scientific literature related to *C. elegans* focuses on individual worm responses and the underlying neural mechanisms, while the analysis of emergent group responses and therefore the social behavior of *C. elegans* remains only partially studied (Ding et al., 2020a). Nevertheless, there lies a huge potential in the possibility of engineering collective responses in *C. elegans* to perform tasks that could be highly beneficial to human beings, such as swarms of *C. elegans* attacking pathogens in crops.

This paper proposes a framework based on terms mainly used in collective behavior to review and categorize studies that investigate the nature of the operational principles driving the collective response of *C. elegans* either through empirical studies or through mathematical and computational models. This categorization helps to determine the ground on which to construct future experimental investigations to identify causal relationships between individual mechanisms and group-level behavioral responses in populations of *C. elegans*. At the same time, we believe that by critically framing this body of literature in *C. elegans* we contribute to identify mechanisms that could be “hacked” with the methods of synthetic connectomics to design swarms of biological animal robots. The framework we propose is organized around three main types of collective responses: aggregation, swarming, and collective decision-making. In our view, these three behaviors can be considered the “principal components” with which to categorize the available research literature and to identify potentially promising future developments in the study of collective behavior in populations of *C. elegans*.

We provide a brief primer of the main modeling approaches discussed in this paper in Table 1. The models described in the following sections are derived from direct observations, thus they valid by construction, albeit only for the phenomenon at hand and often for a limited interval of parameter values.

**Table 1:** Primer of the main modeling approaches discussed in this paper.

**Type**	**Target**	**Scope**
Keller-Segel (Differential Equations)	Population	The model studies how the population density distributes when interacting with one or more chemical cues.
Agent-based	Individual	The model describes how the interaction of individuals among each other and with the environment lead to group behaviors.
Neural network	Neural mechanisms	Computing system inspired by the architecture of brains apt at explaining the neural dynamics of an individual in response to one or more stimuli.

The remainder of the paper is structured as follows. Section 2 gives an overview of the research work focused on aggregation behavior in populations of *C. elegans* ; Section 3 reviews studies on different types of collective and coordinated movements, most of which are directed toward a source of odor (i.e., chemotactic responses). Section 4 gives an overview of those few research works targeting collective decision-making processes in *C. elegans*. In Section 5, we build upon the critical review developed in previous sections to identify fruitful directions for future works to boost our understanding of the mechanisms of collective behavior in *C. elegans*.

## 2 Aggregation

Aggregation is a phenomenon where a group, initially uniformly distributed, reaches a spatial organization in which there exists a set of one or more high population clusters, and another set of one or more spatial clusters with a low, or null, population density (Dias et al., 2021). The *C. elegans* literature on aggregation shows that this group response is regulated by various factors related to genetics, developmental stage, and environmental conditions such as oxygen level, temperature or chemicals concentration, and local worm density. Moreover, we also noticed that different methodological tools (e.g., *in vivo* experiments, mathematical and computer-based simulation models) have been used to promote a relatively dispersed exploration that does not facilitate the development of a thorough understanding of the mechanisms underpinning aggregation in *C. elegans*.

Being one of the simplest possible collective responses and a prerequisite for other more complex social interactions, aggregation is an ineludible candidate to be studied and bioengineered into *C. elegans*. In this Section, we review the studies that have already identified the mechanisms underlying different forms of aggregation in populations of *C. elegans*. *C. elegans* communicate through the release of a class of pheromones termed ascarosides, which were first observed to be attractive to male *N2* and hermaphrodite *npr*−1 worms, while they were repulsive to hermaphrodite *N2* worms (Macosko et al., 2009). A class of derivative pheromones, termed indole ascarosides, promote aggregation among other social signals (Srinivasan et al., 2012). These molecules are composed of four modules: a head group, a carbohydrate, a fatty acid and an acid terminus. Variations of their chemical composition result in different transmitted social signals. For example, variation of a module transforms a repellent indole ascaroside into an attractive one. This library of indole ascarosides could then be used by the worms to interact via a highly developed social communication scheme, a first evidence of multi-layered social signaling in *C. elegans*. A remarkable feature of the indole ascarosides is that they are known to be effective on *N2*, *npr*−1 and the Hawaiian strain hermaphrodite worms at picomolar concentrations. Artyukhin et al. (2018), discovered 86 new ascaroside pheromones, further expanding our knowledge of the vocabulary used by *C. elegans* to communicate. The release of these pheromones depends on the developmental stage and the environmental conditions, although the exact function of some ascarosides remains unknown (Artyukhin et al., 2018). A comprehensive review of nematode pheromones and nematode signaling can be found in Muirhead and Srinivasan (2020) and Yang et al. (2023).

Worms release attractive pheromones upon encountering a specific stimulus, which triggers the aggregation process, as observed in first larval stage (L1) worms. Such scenarios are ideal setups to study and unveil the mechanisms of aggregation, and also to bio-engineer collective responses which would not spontaneously emerge in non genetically modified worms.

Artyukhin et al. (2015) observe that L1 worms form circular aggregates when food scarcity is sensed, particularly when a high number of worms (10^6^ starved worms) are placed in a 6 cm plate containing a low concentration (i.e., 0.01%) of ethanol or any other even-chain alcohol. The morphology of the aggregates then shifts from disk-like to stripe pattern in the course of days, remaining mostly stable in its structure, due to the consumption of ethanol. This is proven by the fact that adding ethanol to a population of worms deployed in a stripe pattern morphology causes the dissolution of the stripe pattern and the re-creation of the circular pattern. Furthermore, the study shows that mutant worms that are unable to metabolize ethanol still aggregate in the presence of wild L1 worms. Hence, the authors speculate that both food scarcity as well as social interactions mediated by some sort of communication signal trigger aggregation. The social interaction induced aggregation is studied in Avery et al. (2021), where the authors develop a mathematical model based on a Keller-Segel system to test the hypothesis that aggregation in L1 worms could be driven by the secretion of a short-range attractant and a longer range repellent pheromone. The disk-like patterns are correctly replicated through numerical solutions of the mathematical model, giving insights into the dynamics governing the aggregation in starved L1 worms. In particular, the model demonstrates that aggregation in food scarce environments can be accounted for by the combination of attractant and repellent pheromones secreted by the larvae. This first example of larval aggregation suggests that this collective response in *C. elegans* is modulated by both attractive and repulsive signals. The use of a Keller-Segel system to model aggregation allows for the inclusion of any number of stimuli, at the cost of losing detail at individual behavioral level. Moreover, these systems require knowledge which is not always available such as details about the functional relationship between the concentration of one or more stimuli and the worm density. Nevertheless, Keller-Segel systems remain a valuable methodological tool to disentangle the causal relationships between multiple stimuli and worm responses.

At adult stage, food presence and population density modulate aggregation patterns observed in *C. elegans*, with significant variations in the group responses in social and solitary strains. de Bono and Bargmann (1998) conduct experiments on the aggregation of *npr-1* (social) and *N2* (solitary) worms in the presence of food. The results of the experiments show that the presence of food and the population density are important factors contributing to aggregation in several species. In particular, in the presence of food, *N2* worms tend to lower their speed of motion and disperse, while *npr-1* mutants move rapidly toward food and form aggregates on the borders of the food patch, a behavior referred to as “bordering” (de Bono and Bargmann, 1998).

In a follow-up study, de Bono et al. (2002) show that bordering is a group response that can be observed in *npr-1* worms under different circumstances, suggesting that it could represent an adaptive response to stressful environmental conditions. Examples of stressful environments include, but are not limited to, food scarcity and unfavorable temperatures.

Bordering is just an instance of a larger set of aggregation responses observed during social feeding. These responses are regulated not only by food that tends to aggregate worms, but also by pheromones that tend to keep the worms away from each other. So far, experiments on group responses in the context of social feeding as in de Bono and Bargmann (1998) and de Bono et al. (2002) have been conducted using a relatively small population size (±80 worms), which may limit the generalizability of the findings to larger populations.

Ding et al. (2019) simulate, using an agent-based model, the behavioral responses of both *npr-1* and *N2* worms during aggregation on food patches (ie., social feeding). Each worm is modeled as an agent undergoing a persistent attractive random walk toward higher local worm densities with a density-dependent reversal rate (i.e., how often an agent reverses its direction of motion). The model shows that the different group responses observed in *npr-1* and *N2* worms when placed in an environment with patches of food can be replicated by modulating reversal rates and the speed of single worm motion with respect to the density of the worms. In particular, to model biological data, *npr-1* worms reverse more often as the local density increases, while *N2* worms do not. On the other hand, both strains have to decrease their velocity as the local density increases, with *npr-1* moving quicker than *N2* worms. Gray et al. (2005) find that the reversal rate of *N2* worms is indirectly proportional to the presence of food: with food scarcity, they tend to reverse their direction.

Another environmental factor that induces aggregation in *C. elegans* is the oxygen level sensed by the worm. Experiments conducted by Demir et al. (2020) found that sub-optimal levels of oxygen (above 12%) induce the formation of aggregates with morphologies resembling Turing patterns in both *N2* and *npr-1* worms. Moreover, these experiments reveal that the presence of food does not alter the aggregation dynamics, suggesting that the levels of oxygen have a stronger effect on worm behavior than availability of food. The authors develop a macroscopic Keller-Segel model which allows to predict aggregation by imposing boundaries on the density of worms, which in turn depends on the initial oxygen levels. The model suggests that aggregation in both strains happens in the presence of suboptimal levels of oxygen and a relatively high population density, providing numerical bounds for the population density given a specific oxygen level. However, the model suffers from a numerical limitation on the upper bound of the worm density: if the density is above this bound, the model predicts that no aggregation occurs, in contradiction with findings that leverage an agent-based model undergoing a persistent random walk (Vellinger et al., 2024).

Several models are developed to test hypotheses on the nature of worms individual mechanisms underlying aggregation. For example, at the macroscopic level, the aggregation process in a high density population of *C. elegans*is expressed as a phase separation process in Chen and Ferrell (2021). The authors describe the colony formation (i.e., aggregation) of the worms as a condensation phenomenon, where the formation of a cluster breaks the symmetry of the system, leading to high and low density areas. This process is modeled with a single ordinary differential equation, where the key parameter for the stability of the aggregate is given by the amount of worms entering and leaving the aggregate itself. The limitation of such a model resides in that it depends on a parameter which is extrapolated from observations, without any rule to predict it based on external conditions; essentially, it lacks predictive properties.

Conversely, a microscopic (agent-based) model based on active physics for the dynamical network aggregates in *C. elegans*is proposed by Sugi et al. (2019). In this model, the main factors influencing the aggregation process are the alignment of worms after collisions and smooth turning. The model takes into account a repulsive and an attractive force generated by the excluded volume of worms and by the surface tension of water around worms, respectively. The microscopic model successfully reproduces the dynamical network formation and its dependence on experimentally controlled parameters, providing a minimal physical description of *C. elegans* collective motion within the framework of active matter physics. However the results indicate that the model is unable to accurately reproduce the behavior of the worms. It is argued that this is due to factors such as individual trajectories not being taken into account. Nevertheless, the inclusion of these features could significantly increase the complexity and the computational resources required to run the model, thereby hindering the use of the model for large populations.

The physical medium on which worms move influences their locomotion: they swim in liquids and crawl on solid surfaces. When swimming in a liquid, it has been observed that worms within an aggregate are able to synchronize their gait (Yuan et al., 2014). The model suggests that the main factor influencing gait synchronization is the distance between worms. In particular, worms that are closer to each other tend to synchronize their gait more frequently than those far apart. The model approximates the physical embodiment of the worm with a fine-grained level of details that renders the simulation of large size populations computationally expensive. The experiments carried in Yuan et al. (2014) feature few worms, leaving out the possibility to investigate how density-induced phenomena could interact with the synchronization induced effect. A similar model to Yuan et al. (2014) was developed few years before by Niebur and Erdös (1991). In this early model, the authors consider the dynamics of groove formation on the drag forces. The formation of grooves under the body of the worm in different environmental conditions, such as wet agar or a water film, modifies the lateral forces the worm can exert to propel itself. The model shows the physics behind the locomotion of worms, arguing that the latter are governed by the surface on which the worms move, which ultimately plays a role in the likelihood of gait synchronization and thus the dynamics of worms inside aggregates. We hypothesize that worm gait synchronization could bring forward coordinated movement among worms. Thus, the use of a specific medium, liquid or solid, given the knowledge of how the medium influences the likelihood of gait synchronization, could play a vital role in engineering specific responses. Moreover, the morphological properties of the environment induce the emergence of group responses in *C. elegans*. For example, the presence of a vertical structure, such as a toothbrush bristle, in a high worm density environment without food induces the worms to form tower-like structures. It was reported that *C. elegans* worms are able to form a 3 dimensional tower as a mechanism for dispersal (Perez et al., 2025). This structure is adopted both in the wild and in laboratory settings, across multiple strains and developmental stages, without a clear specialization of individuals within towers. The tower-like aggregate is able to move on its own, extending the arms toward passing objects and subsequently dispersing. It is also responsive to touch, which implies that mechano-sensation plays an important role. In the context of bioengineering animal neural circuits, understanding the neural basis of tower formation could help in designing neural modifications that induce this phenomenon in a desired setting.

Overall, the literature on *C. elegans* aggregation is composed of macroscopic and microscopic models. The macroscopic models are characterized by sets of differential equations, such as Keller-Segel models (Demir et al., 2020; Avery et al., 2021) that describe the interaction of a population density with one or more chemicals or simpler models with a single equation that describes the rate at which worms leave an aggregate (Chen and Ferrell, 2021). These models are useful for describing the interaction mechanisms of large populations of *C. elegans* although a mean field approach requires knowledge about how exactly worms integrate multiple cues and how this integration brings forward specific behaviors. For this reason, agent-based models (Niebur and Erdös, 1991; Yuan et al., 2014; Ding et al., 2019; Sugi et al., 2019; Vellinger et al., 2024) are necessary to gain knowledge of the response of a single worm to one or more stimuli, which can then be expanded to include the effect of other worms. With this spirit, the following sections contain the basic blocks for understanding the operational principles underpinning single worm responses in scenarios which are extensible to large populations.

## 3 Swarming

Swarming refers to group movements of a population of agents toward a common direction, usually in a coordinated fashion and stemming from a collective decision, with aligned velocity vectors. The coordinated response of a group of bioengineered *C. elegans* could have numerous applications. For example, a group of worms might be programmed to swarm toward a bacterial patch and, equipped with a strain-specific bacteriophage virus, destroy it. The literature on swarming in *C. elegans* is relatively limited. In those few studies focused on swarming, the group movement is generated in response to chemical stimuli. Thus, swarming is generally referred to as collective chemotaxis. It has been observed that groups of *C. elegans* also swarm toward patches of bacterial food (Ding et al., 2020b). In particular, worms first aggregate on a food patch and, once food is consumed, they collectively move toward another patch. In the presence of multiple food patches with varying density, worms distribute proportionally to the density of the food patch (Madirolas et al., 2023). This proportional distribution is observed for food patches made of different bacterial strains, although its mechanisms remain unexplored. It is assumed that the release of different types of pheromones contributes to the distribution of worms among the food patches, balancing the local worm density (Dal Bello et al., 2021; Luo and Portman, 2021; Molina-García and Barrios, 2021). A similar mechanism was observed during chemotaxis in Matsuura et al. (2005), where experiments show that the relative amount of *N2* worms reaching the source of an attractive chemical stimulus (measured by the chemotaxis index) is inversely correlated to the population density. The authors account for this evidence by formulating the hypothesis that the release of repellent pheromones by worms hinders the development of a group movement toward the stimulus. The repellent pheromones are produced to alert other worms that a particular attractive odor, usually associated to food, is being (or will be soon) depleted. Thus, this pheromone production could have evolved to regulate the population density of *C. elegans* at a food location.

An agent-based model of chemotaxis based on the pirouette rate is described in Pierce-Shimomura et al. (1999). This model is able to qualitatively and quantitatively reproduce the pirouette behavior of *C. elegans* during chemotaxis assays with attractive odors. The authors provide a detailed description of operational principles accounting for the development of chemotaxis in individual *C. elegans*. In particular, the concentration of the attractive stimulus modulates the worms' pirouette rate, which is argued to be the main strategy worms use to explore their environment. The worm movement during chemotaxis is decomposed into two behavioral states: one characterized by mostly straight runs, by which the worms exploit their “knowledge” of the odor gradient in order to climb it; the other, consisting of sharp reorientations, by which the worms explore the environment to avoid points of odor local minima. The exploration is performed via a three-stage process. First, worms compare the current level of the chemical concentration with what they previously experienced, a process termed differentiation. Levels above a certain sensory threshold are attenuated (low-pass filtering) and a non-linear function is applied to the currently sensed gradient to obtain the pirouette probability.

The influence of pirouettes in *C. elegans* during chemotaxis is also the subject of the work in Yoshimizu et al. (2018), which shows that the chemotactic behavior of worms moving toward a food source differs depending on whether the worms are alone or with other worms. In accordance to what was previously shown in Matsuura et al. (2005), the results discussed in Yoshimizu et al. (2018) suggest that *N2* worms releases some repulsive pheromone which influences the pirouette rate of the other worms when climbing a chemical gradient. In particular, in single-worm studies, *N2 C. elegans* tends to directly climb the gradient. When operating in groups or even in pairs, *N2 C. elegans* first disperse with pirouette behavior and only later move toward the attractant source.

It is also noted that single worms have a higher probability of performing pirouettes at lower concentration of the attractive cue than those in groups and pairs. As in other similar studies, the relatively small group size (max 8 worms) hinders the possibility to observe and correctly evaluate the extent to which the group density influences pirouette initiation, dispersal and consequently chemotaxis.

Ding et al. (2020a) address strain-specific behavioral differences in collective foraging. In particular, they perform a comparative study of the specific relationships betweens the behavior of solitary (i.e., *N2*) and social (i.e., *npr-1*) strains of *C. elegans* and the distributions of food in a self-regenerating food environment developed in Bhattacharya and Vicsek (2014). The results of this study suggest that the foraging of the two strains depends on the food distribution: the solitary *N2* strain is more efficient in an environment containing uniformly distributed food, rather than patchily distributed food. In the social *npr-1* strain, the opposite holds true. The experiments also indicate that the *N2* strain is always more efficient than *npr-1* in consuming food. Nevertheless, this conclusion applies only to the relatively small groups (≈ 40 worms) used in the study. In larger groups density-induced responses of social feeders may enhance their foraging efficiency and consequently reverse the conclusions of this study.

Pandya et al. (2015) build an agent-based model looking at *C. elegans* chemotaxis toward bacteria which release an attractive odor and, once ingested, kill the worms through the release of a toxin. Experiments with 100 *N2* worms are performed to analyse the correlation between the chemotaxis index and (i) the diffusion constant, which dictates the rate of diffusion of the attractive odor, and (ii) the strength of the attraction, which dictates how strongly worms respond to an attractive odor.

In the microscopic model, the heading direction is influenced by the direction toward the bacterial colony and the perceived strength of attraction, while the velocity is drawn from a normal distribution based on the experimental data. In a previous study, Zhang et al. (2005) have shown that after a short exposure to a toxic bacteria, worms are able to learn to subsequently avoid it. Thus, the model is expanded by allowing the agents to learn about the toxicity of the attractive source and consequently decrease their probability of heading toward it, through an association between food toxicity and attractive odor. Furthermore, the authors analyse the response of the model when the odor is not static, but dynamically fluctuates with a periodic pulse. They find that longer pulse periods are associated with a lower chemotaxis index. Overall, the model predicts a consistent decrease in the chemotactic index when agents are able to learn about the toxic substance across values of the diffusion constant and of attraction strength.

While Pandya et al. (2015) focus on the collective behavior and density-dependent chemotaxis of *C. elegans*, Soh et al. (2015) take a different approach by building an agent-based model for the simulation of the full body movement of *C. elegans* during chemotaxis. They start from a model of a robot snake to model the motion of the full body of a worm through a Newton-Euler system. This system allows to model the translational and rotational dynamics of a rigid body through a set of differential equations for the position and the torque of a rigid body (Hahn, 2002). Additionally, they model the information-processing of *C. elegans* where a set of second-order differential equations governs the output of sensory neurons responsible for the detection of the attractant odor. The output of the sensory neurons is then fed to a neural network composed of three layers of interneurons, which finally outputs the probability of performing a specific movement selected from pirouette, the weathervane mechanism (a curving of the body toward the highest concentration of the odor during forward motion), and random walk. The results indicate that the gradient of the attractive source could be determined based on the angle of the head, where the sensory neuron for the attractive odor is located, and the second-order differentiation of the chemical gradient. However, their parameter tuning was done manually and thus may not represent the natural *C. elegans* features. Moreover, it considers the model of a single worm, thus its relationship with density-dependant behaviors is not explored.

Matsuoka et al. (2008) construct an agent-based model with Monte Carlo simulations for the thermotaxis i.e., the movement influenced by temperature, for large (>25, 000 worms) populations of *C. elegans*. Their simple model confirms results already obtained in previous studies where the cultivation temperature of worms influences their preferences for temperature. In particular, worms presented with a thermal gradient below their cultivation temperature move more randomly than when presented with a gradient above the cultivation temperature.

*C. elegans* can be sensitive to the level of humidity, displaying hygrotaxis (Russell et al., 2014). The preferred level of atmospheric moisture in adult worms depends on their experience during the developmental phase. In particular, if they experience lack of food and high humidity levels, at adult stage they are attracted to lower levels of humidity.

Furthermore, Parida and Padmanabhan (2016) report that *C. elegans* are able to perform durotaxis, i.e., to move in response to the stiffness of the underlying surface. In particular, worms are attracted to stiffer regions. This holds true even when a humidity difference is present, although the effect of moisture dominates the effect of stiffness when the difference in stiffness of the regions is low. Moreover, in the presence of a chemical attractant, worms climb the chemical gradient without preference for the surface stiffness. Overall, hygrotactic and durotactic assays provide insights into the response of *C. elegans* to varying environmental conditions, which could potentially influence the response of worm collectives and thus should be kept in mind for the design of biological animal robots.

The relatively limited number of models on swarming tends to exploit agent-based models as a methodological tool for representing individual responses to attractive or repulsive stimuli (Pierce-Shimomura et al., 1999; Matsuoka et al., 2008; Pandya et al., 2015; Soh et al., 2015). The principles of *C. elegans* swarming are less understood than those underpinning aggregation. The reason behind this is that the environment in aggregation scenarios is globally isotropic, meaning that the stimuli are globally uniformly distributed, while local gradients are induced by, e.g., oxygen consumption. On the other hand, in swarming scenarios, the environment is globally not isotropic. For example, food patches need to be non-uniformly distributed for worms to leave them by swarming toward another patch, and an odor gradient must be present for worms to climb it. Moreover, common laboratory strain *N2* worms are inadequate for the development of a swarming group response as the effect of their pheromones tends to induce dispersion. Thus, one plausible direction for bioengineering swarming *C. elegans* could be the modification of the neural circuit responsible for producing an aversive response to pheromones in *N2* worms. Whether it is more adequate to completely suppress the aversion or to modulate it, either to a less aversive or to an attractive response, requires further investigation.

## 4 Collective decision-making

A decision-making process is referred to as collective once none of the actors of the process can be considered responsible for the final decision. Experimental studies show that worms are able to make decisions on the fly by integrating multiple, possibly contradicting, environmental cues. For example, once on a particular food patch, the decision of a worm to remain or to leave the patch is based on considering sensory cues that generate opposite responses, since the food itself can generate both an attractive and a repulsive reaction (de Bono et al., 2002). In these circumstances, the final decision depends on the particular strain at hand (Bendesky et al., 2011).

As shown in Tanimoto and Kimura (2019), behavioral choices determined by conflicting chemical signals require the neural accumulation of antagonistic cues either through recurrent synaptic circuits responsible for sensing these cues (Gold and Shadlen, 2007) or through intracellular mechanisms (Curtis and Lee, 2010). The neural mechanisms underlying this type of decisions can be unveiled by analyzing (i.e., by isolating and observing the activity of) the neural sub-modules responsible for generating the possible responses starting from a particular set of stimuli (Tanimoto and Kimura, 2019). For example, Ishihara et al. (2002) place a stripe of repulsive odor between the initial position of the worms and an attractive odor. Their findings show that wild isolates cross the stripe, whereas *N2* worms, in general, do not, suggesting different mechanisms for the integration of the environmental cues. Another example is given by the neural network for chemotaxis constructed in Appleby (2012). The resulting circuitry is simpler than the one possessed by worms, although able to reproduce chemotaxis in a biologically plausible way. The more intricate circuit of *C. elegans* is responsible for the integration of multiple, possibly overlapping, sensory cues into a common downstream circuitry.

The principle of integrating multiple sensory cues applies also to foraging *C. elegans*. As they consume the food patch, they alternate between two behavioral states: roaming, which typically occurs when a worm leaves a patch in search for a better one, and dwelling, characterized by a local exploitation of the patch. The choice to leave the patch is mediated by numerous stimuli, such as satiety and chemical odors (Ji et al., 2021). For example, worms adjust their search strategy based on the food distribution encountered within the last half an hour (Calhoun and Hayden, 2015). Moreover, the neurons responsible for the transition between the roaming and dwelling states are known (Ji et al., 2021). Furthermore, it has been observed that the choice to leave a food patch happens mostly during the roaming state. In particular, worms briefly accelerate right before leaving the patch (Scheer and Bargmann, 2023). A sensory cue responsible for the transition between the roaming and dwelling state is given by pheromones, signaling the presence of competitors (Molina-García and Barrios, 2021). For example, as discussed in Section 2, ascaroside pheromones are mostly repulsive for hermaphrodite *N2* worms. However, in the presence of food, the repulsion diminishes (Molina-García and Barrios, 2021). The modulation from attraction to repulsion is present only in hermaphrodites and is mediated by the abundance of food (Dal Bello et al., 2021; Molina-García and Barrios, 2021).

The specific response of a worm, whether repulsive or attractive, given by a stimulus is flexibly represented within the neural circuit by encoding past experiences and predictions of that stimulus (Molina-García et al., 2024). An example is that worms are innately attracted to salt; however, through aversive conditioning, the worm learns to avoid salt. When pairing a positive stimulus to a negative experience, two memories compete for behavioral expression.

Tanimoto et al. (2017) provide a simple state-machine (i.e., a type of model used to describe the behavior of a system in terms of its states, transitions, and actions) for the odor avoidance in *C. elegans*, where, in accordance to what discussed in Pierce-Shimomura et al. (1999), worms alternate between a state of pirouettes and a state of straight runs, depending on the concentration of the repellent. The pirouette and the run states are similar in terms of behavioral output to the dwelling and the roaming states, respectively. This parallelism suggests that even if the behavioral repertoire of the animal is limited in terms of states, these are adaptable with respect to the environment. In particular, the adaptation is represented by the modulation of the transitions from a state to another, together with modifications to the heading in such a way that the best direction can be followed. The transition from pirouette to run is dictated by the time integral of the change in repellent. The worms require an accumulation of the antagonistic cue before they switch to the run state, whereas the switch from run to pirouette only takes place in response to variations in repellent: when it is positive, it means the worm is going toward the repellent, so it needs to quickly readjust its heading by means of pirouettes (see also Figure 5 in Pierce-Shimomura et al., 1999).

Haley and Chalasani (2024) provide a broad view of the foraging strategies in *C. elegans* interpreted as a decision-making process. In particular, they provide a useful schematic diagram that provides a holistic view of all the decisions related to foraging in *C. elegans*, from exploration to exploitation, including dietary preferences and food distribution.

In their assay, Ghosh et al. (2016) place a ring of hyperosmotic fructose around the initial position where worms are placed on the agar plate. Their result indicate that a low proportion of worms (≈30%) leave this ring, but when two spots of attractive dyacetil are placed further from the ring, this proportion increases significantly (80%). Motivated by these results, they uncover a neural circuit that regulates the integration of threats and rewards in *C. elegans*, revealing a neural architecture for how these decisions are made.

Further experiments are required to reveal whether group effects, mediated by pheromone signaling, might also play a role in the decision to initially disperse from the ring. More generally, if we were to induce swarming into bioengineered worms as per Section 3 and to combine it with the decision-making principles of Ghosh et al. (2016), we could design a multi-level system of biological robots. Such biological robots could first aggregate in a spot, then through a carefully designed decision-making process, select another spot to swarm to. This would represent a first example of a completely autonomous biological swarming robot capable of finding and destroying bacterial patches. However, such a design requires careful consideration of the possible behaviors a worm might favor depending on the environmental conditions at hand. Other than chemical odors, another common environmental cue is food, which induces specific behaviors.

Scholz et al. (2017) construct a Markov model for the stochastic feeding of *C. elegans*, which consists of rapid bursts of pumping food from the environment followed by states in which they pause their feeding. They performed assays with different food concentrations and found that worms stochastically switch from sampling their environment, integrating information from the environment, to either committing to feeding in rapid bursts, or to pausing. The probability of switching could be modeled on a Bayesian inference process, where the decision taken by a worm depends on its estimate of a probability distribution. The results suggest that there exists a trade-off between the speed and the accuracy with which worms are able to pump food. Another trade-off exists between exploration and exploitation, where worms try to maximize their intake with past knowledge about their environment, without sacrificing their adaptability to dynamic environments.

These results, coupled with previous studies, show that past experiences, environmental cues, developmental stage and sex-specific preferences shape the behavioral output of the worm. In particular, all these factors are integrated within the neural circuit of the worm, where numerous sensory neurons propagate signals to a common downstream muscular circuitry (Macosko et al., 2009). Understanding the operational principles of this biological bottleneck could help to establish the rules that govern decision-making in *C. elegans*. In the remainder of this section, we present several *C. elegans* neural circuits that have been analyzed and reconstructed *in-silico*.

Sarma et al. (2018) present the OpenWorm project, which aims at building a framework that allows the simulation of the biophysical neural activity and of the biological tissue of *C. elegans*. Their goal is to develop a framework capable of explaining how the behavior of the worm arises from its biological features. In particular, they employ a level of detail that requires relatively high computational power in order to simulate the behavior of *C. elegans*. Although it is possible to select different simulation engines which are more or less detailed, to date, some functionalities of the framework do not correspond to the biological behavior of the actual worm. The inherent complexity of such a simulator renders it heavily dependant on the hardware used to run the simulations and possibly unusable for the simulation of multiple worms, let alone for a swarm.

The complexity of reconstructing the entire worm connectome resides in that we know its structure (where neurons are placed), but we lack the knowledge regarding its functionalities. For example, the sign (inhibitory or excitatory) and strength of some synapses is unknown. Furthermore, neurotransmitters and extrasynpatically released neuropeptides play a crucial role in determining the functionality of the worm neural network, inducing functional variability across worms due to neural plasticity and diverse past experiences (Randi et al., 2023). A possible direction for clarifying the relationship between structure and functionality in the *C. elegans* connectome could be to abstract the neural sub-modules responsible for specific behavioral responses to artificial neural networks. For example, some connectomic-oriented approaches to construct models inspired by the *C. elegans* connectomes have been explored by Yan et al. (2022), demonstrating the effectiveness of sparse backpropagation algorithms in creating bionic structures that mimic the *C. elegans* nervous system. Although it is not confirmed if *C. elegans* employs a mechanism akin to backpropagation, its neural activities show similarities to recurrent neural networks (RNNs) (Hassabis et al., 2017; Della Vecchia, 2021). Hernandez et al. (2022) explored Spiking Neural Networks (SNNs) to develop a *C. elegans* inspired model. These connectome-inspired models often lack structural bio-plausibility, but deliver adequate performances in their given tasks.

A number of works rely on the optimisation of neural networks that model the nature of the synapses located in neural sub-circuits of *C. elegans*. Cangelosi and Parisi (1997) describe how they trained a neural network to simulate the circuit responsible for the touch response in *C. elegans*using a genetic algorithm. Similarly, Lanza et al. (2021) build a recurrent neural network (RNN) for the response of *C. elegans*to aversive stimuli using Monte Carlo simulations. Barbulescu et al. (2023) train a RNN, a long-short term memory (LSTM) unit and a gated recurrent unit (GRU) in order to simulate the whole connectome of the worm, finding that the latter is able to best simulate and adapt the network dynamics against a wide range of stimuli. In order to test their models, they use data generated from the c302 model of *C. elegans* (Gleeson et al., 2018), the same framework leveraged by the OpenWorm project, in particular using the NEURON simulator (Carnevale and Hines, 2006). A similar approach is used by Roberts et al. (2016) for the development of a stochastic state machine representing the synaptic circuit responsible for the search behaviors of *C. elegans*. They find four possible internal states for the worm: a forward and a reversal of the movement and two states where they do not move. The probability of switching between these states is found from molecular level mechanisms of the neurons responsible for the movement of the worm. Their findings suggest that the most likely state for a worm is going forward, followed by a pause of movement, which can lead to an equiprobable switch to the forward or the reversal state. Finally, the reversal state almost exclusively leads to a pause state, which leads almost exclusively to the forward state.

Izquierdo and Lockery (2010) found a minimal neural network that models the klinotaxis of *C. elegans*through an evolutionary algorithm which not only replicates correctly the klinotactic behavior, but also displays novel orientation behaviors which arose as emergent behaviors of the network. The results provide useful insights in the understanding of the neural circuits which govern the klinotaxis of *C. elegans*. Similarly, Matsumoto et al. (2024) find a neural network model for the navigation of *C. elegans*: worms integrate sensory knowledge about the local concentration of the odor and motor information on the bearing angle of their neck, thus leading to klinotaxis.

Another approach for modeling the whole nervous system of *C. elegans* can be found in Pavlovic et al. (2014), where authors utilize a Erdös-Rényi Mixture Model (ERMM) to find blocks (or modules) of neurons which are related to the same functional circuit. Indeed, providing functional blocks gives a greater degree of explainability with respect to single neurons. Their approach consists in detecting neural clusters which have a high internal connectivity and a lower connectivity with other clusters. The authors find that the stochastic ERMM yields 9 blocks, compared to 4 − 5 blocks in deterministic models. Overall, the blocks correspond between the two methods, but ERMM is able to provide a biologically more accurate block division, allowing to compress the connectome of *C. elegans*. This approach shows a promising path for the simulation of swarms of *in-silico* worms.

In summary, when populations of *C. elegans* integrate conflicting stimuli (e.g., in a multiple options decision-making scenario), individual factors coupled with environmental cues and population-level dynamics contribute to determine the decision of the collective. In particular, neural circuits handle antagonistic signals, sensory inputs (e.g., chemical gradients, temperature shifts) shape the worm's immediate preference, and inter-individual factors like crowding or pheromone-based signaling contribute to the emergence of the final collective decision.

## 5 Discussion

We have opened this paper by introducing a novel approach to the synthesis of artificial biological creatures based on the methods of neuro-synthetic biology. We mentioned that synthetic connectomics could potentially pave the way to a novel form of direct engineering for the design of biological creatures (i.e., biological animal robotics). In the design of biological animal robots, the individual consists of a biological organism, naturally evolved over millions of years, containing extensive synthetic modifications to its nervous system intended to redefine its behavior. The design principle underlying such biological, carbon-based robotics is similar to that of modern silicon-based robotics: the modified neural circuits act as a control unit, harnessing the naturally-occurring wetware of biological animals, thus overcoming the limitations of silicon-based hardware in terms of adaptation and robustness in natural biological environments. *C. elegans* being the first animal with a fully mapped connectome, and with a relatively short developmental phase, represents a highly beneficial biological platform for exploring the potential of biological animal robotics. In particular, populations of *C. elegans* could be designed to operate in a cooperative and coordinated way to carry out tasks that are beyond the competencies of single worms.

The engineering of collective responses in populations of *C. elegans* would benefit from a solid understanding of the mechanisms regulating the natural worm responses in high-density populations under varying environmental conditions. Our review points to the fact that only a minority of *C. elegans* literature focuses on population dynamics and the mechanisms underpinning collective behavior in *C. elegans*. These few studies use different methodological approaches ranging from classic *in vivo* experiments, to microscopic (i.e., agent-based) and macroscopic models implemented with systems of differential equations. However, this interdisciplinary approach has so far generated a still fragmented picture of *C. elegans* collective behavior.

We reviewed the literature on collective behavior in *C. elegans* with respect to three fundamental social responses (i.e., aggregation, swarming, and collective decision-making). With the aim to identify fruitful directions for future works, we superpose to this three-behavior framework another framework, originally illustrated by Nolfi and Floreano (2000), where the mechanisms underlying collective responses are divided in three categories based on the level (i.e., supra-organism, organism, and sub-organism) at which they operate.

The supra-organism level, corresponding to the observed collective response (e.g., aggregation, swarming, collective decision-making), includes mechanisms followed uniformly by all individuals. These mechanisms are generally modeled and investigated using systems of differential equations. The organism level includes mechanisms that govern the interactions among individuals and between the individual and the physical environment through chemical, mechanical, and thermal cues. These mechanisms are generally modeled and investigated using agent-based models. Finally, the sub-organism level includes mechanisms that modulate individual responses by taking into account the individual physiological factors such as developmental stage, sex, past experiences and the strain at hand. These mechanisms are investigated by developing models that look at the neural dynamics responsible for the worm motor output.

**Figure 1:**
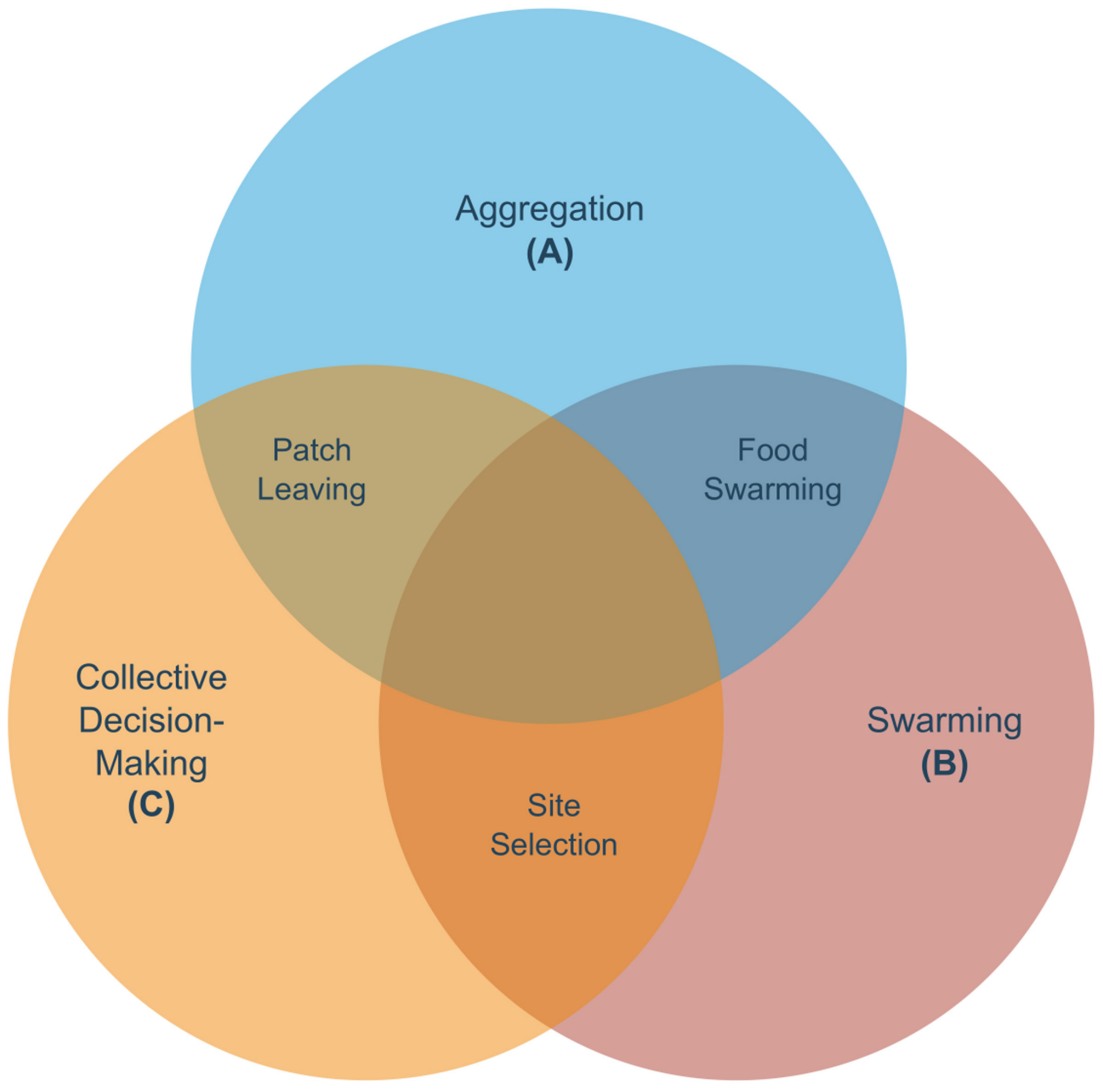
Venn diagram composed of the three main collective responses analyzed in this review. Each collective behavior is categorized into a set, while the intersections represent observed or hypothesized composite behaviors in *C. elegans*. The intersection of aggregation and swarming is represented by food swarming, where worms first aggregate on a patch, consume it, and swarm toward a more prosperous patch. The intersection of swarming and collective decision-making is hypothesized to be akin to a site selection task, where worms need to select collectively a patch and swarm toward it. To the best of our knowledge, this has not yet been observed in collectives of *C. elegans*. The intersection of aggregation and collective decision-making contains patch-leaving: aggregated worms need to choose whether to leave the patch.

The Venn diagram in Figure 1 shows the three fundamental collective behaviors and other social responses (hereafter, referred to as derived) that we have identified as resulting from any combination of the fundamental collective behaviors. Table 2 shows how all the fundamental and derived social behaviors fit into the three-level framework and the related work that has been already dedicated to each of them. At the interaction between aggregation and swarming, we placed food swarming (see Figure 1), a process where worms first aggregate on a patch, consume it, and subsequently swarm toward a more prosperous patch. Table 2 tells us that this phenomenon has been studied at the organism and sub-organism level by Ding et al. (2020b) and Sawin et al. (2000). At the interaction between swarming and collective decision-making we placed site selection (see Figure 1), a process where worms need to collectively select a patch and swarm toward it. An instance of site selection has been studied at the supra-organism level by Madirolas et al. (2023), although experiments make use of only 5 worms, and at the sub-organism level by Izquierdo and Lockery (2010), Appleby (2012), Roberts et al. (2016), and Matsumoto et al. (2024). The dynamics of organism level phenomena in site selection tasks remain under explored and further studies are required to link the supra and sub-organism levels for this response. At the interaction between aggregation and collective decision-making we placed patch-leaving (see Figure 1), where aggregated worms need to choose whether to leave the current patch or remain on it. Patch-leaving has been studied only at the sub-organism level (de Bono et al., 2002; Ji et al., 2021; Scheer and Bargmann, 2023). Finally, we hypothesize that the intersection of the three sets might contain compound social responses, allowing a combination of the three responses. To the best of our knowledge, studies on compound social responses in *C. elegans* are still non existent, thus this column is omitted from the table. The empty cells of Table 2 represent future directions for further developing the field of collective behavior in *C. elegans*. For example, there exist studies pertaining to the supra-organism level aggregation and site selection sets, but not to the other sets. The absence of high-level descriptions for swarming and collective decision-making shows that the population-level dynamics of these two responses remain poorly understood. Similarly, food swarming and patch leaving miss a supra-organism level representation.

**Table 2:** Table of publications cited in this review organized.

**(III)**	Avery et al., 2021; Demir et al., 2020; Chen and Ferrell, 2021; Perez et al., 2025					Madirolas et al., 2023
**(II)**	de Bono et al., 2002; Ding et al., 2019; Vellinger et al., 2024; Sugi et al., 2019; Yuan et al., 2014; Niebur and Erdös, 1991	Matsuura et al., 2005; Pierce-Shimomura et al., 1999; Yoshimizu et al., 2018; Ding et al., 2020a; Pandya et al., 2015; Matsuoka et al., 2008	Tanimoto et al., 2017; Haley and Chalasani, 2024; Scholz et al., 2017	Ding et al., 2020b		
**(I)**	Yang et al., 2023; Muirhead and Srinivasan, 2020	Soh et al., 2015	Tanimoto and Kimura, 2019; Ishihara et al., 2002; Molina-García and Barrios, 2021; Molina-García et al., 2024; Ghosh et al., 2016; Macosko et al., 2009; Sarma et al., 2018; Randi et al., 2023; Yan et al., 2022; Della Vecchia, 2021; Hassabis et al., 2017; Hernandez et al., 2022; Cangelosi and Parisi, 1997; Lanza et al., 2021; Barbulescu et al., 2023; Gleeson et al., 2018; Carnevale and Hines, 2006; Pavlovic et al., 2014	Sawin et al., 2000	de Bono et al., 2002; Milward et al., 2011; Ji et al., 2021; Scheer and Bargmann, 2023	Appleby, 2012; Roberts et al., 2016; Izquierdo and Lockery, 2010; Matsumoto et al., 2024
**A**	•	°	°	•	•	°
**B**	°	•	°	•	°	•
**C**	°	°	•	°	•	•

The top part divides the publications into three levels: supra-organism (**III**), organism (**II**) and sub-organism (**I**). The bottom part shows the possible combinations of collective responses starting from the three axis of aggregation (**A**), swarming (**B**) and collective decision-making (**C**). Black and white dots indicate if a set is selected or not, respectively.

We believe that this relatively “patchy” state-of-the-art description of mechanisms underlying collective behavior in populations of *C. elegans* requires a more systematic research work possibly supported by synergistic contributions from different methodological tools. We suggest that experimental work should focus on discerning the effects of pheromones from those of population density. This could be done, for example, by observing and analyzing the behavior of worms that are deficient in pheromone production/sensing (e.g., *daf-22* mutans), within increasingly higher population densities in order to compare their behavior to that of worms that produce pheromones (e.g., *N2* or *npr-1* mutants). Such comparative experiments could exploit new techniques to automate tracking of single worms and to analyse their behavior, in order to better understand how density influences the behavior of worms and what effect pheromones have in these high density aggregates. The use of an interdisciplinary methodological toolkit could help in overcoming practical limitations of our understanding of the operational principles governing the release of pheromones in varying environmental and social conditions. For example, it could provide models and *in-silico* experiments of biologically plausible responses to different types of stimuli. These models could exploit *in vivo* elements, such as the tracking data of *C. elegans*, to validate “data-driven” assumptions made on the dynamics governing the release of pheromones in specific environmental conditions. Multiple types of descriptive data can be collected by odor chambers (Chen et al., 2023) for populations of *C. elegans* navigating odor gradients, potentially leading to the development of models of the social interactions during swarming. Further and more complex models could be used to validate assumptions on the interactions of multiple causal factors of collective behavior. Important factors are those related to the release of and response to pheromones and those related to the dynamics governing the reaction to mechanical social stimuli potentially leading to gait synchronization.

To conclude, we believe that scientific progress on the understanding of collective behavior of populations of *C. elegans* requires an extensive analysis of data relative to the movement of worms in increasingly higher density scenarios. Moreover, the field would benefit from the development of data-driven models, which need to be validated to provide insights into the causal rules linking individual actions, neural mechanisms, environmental conditions, and emerging collective responses. A theoretical and empirical understanding of the principles driving natural collective behavior in *C. elegans* could allow us to instill them with specific synthetic collective behavior through neural and synaptic engineering. At the same time, engineering synthetic collective behavior could serve as a powerful method for uncovering lower-level mechanisms underpinning collective behavior, effectively bridging the gap between the functionality and the connectivity of the connectome, providing a bottom-up description of collective behavior.
